# Microbial Nitrogen-Cycle Gene Abundance in Soil of Cropland Abandoned for Different Periods

**DOI:** 10.1371/journal.pone.0154697

**Published:** 2016-05-03

**Authors:** Huhe, Shinchilelt Borjigin, Buhebaoyin, Yanpei Wu, Minquan Li, Yunxiang Cheng

**Affiliations:** 1 Institute of Soil and Fertilizer and Save Water Agricultural, Gansu Academy of Agricultural Sciences, Lanzhou, Gansu, China; 2 Graduate School of Life and Environmental Sciences, University of Tsukuba, Tsukuba, Ibaraki, Japan; 3 Graduate School of Science, Chiba University, Chiba, Japan; 4 State Key Laboratory of Grassland Agro-ecosystems, College of Pastoral Agriculture Science and Technology, Lanzhou University, Lanzhou, Gansu, China; 5 Gansu Academy of Agricultural Sciences, Lanzhou, Gansu, China; Universiteit Utrecht, NETHERLANDS

## Abstract

In Inner Mongolia, steppe grasslands face desertification or degradation because of human overuse and abandonment after inappropriate agricultural management. The soils in these abandoned croplands exist in heterogeneous environments characterized by widely fluctuating microbial growth. Quantitative polymerase chain reaction analysis of microbial genes encoding proteins involved in the nitrogen cycle was used to study *Azotobacter* species, nitrifiers, and denitrifiers in the soils from steppe grasslands and croplands abandoned for 2, 6, and 26 years. Except for nitrifying archaea and nitrous oxide-reducing bacteria, the relative genotypic abundance of microbial communities involved in nitrogen metabolism differed by approximately 2- to 10-fold between abandoned cropland and steppe grassland soils. Although nitrogen-cycle gene abundances varied with abandonment time, the abundance patterns of nitrogen-cycle genes separated distinctly into abandoned cropland versus light-grazing steppe grassland, despite the lack of any cultivation for over a quarter-century. Plant biomass and plant diversity exerted a significant effect on the abundance of microbial communities that mediate the nitrogen cycle (*P* < 0.002 and *P* < 0.03, respectively). The present study elucidates the ecology of bacteria that mediate the nitrogen cycle in recently abandoned croplands.

## Introduction

The Inner Mongolian steppe has long been used by pastoral nomads. However, for the past 40 years, rapid population increase across the steppe grasslands has resulted in the nomadic society yielding to semi-agricultural and semi-grazing lifestyles. In some areas, inappropriate agricultural management caused soil degradation and desertification to such an extent that croplands were abandoned [1, 2]. In response to this situation, the Chinese government began a plant reintroduction program with the goal of restoring such areas to grasslands or forests [3]. In addition to China [4–6], such programs have been introduced in Sweden, United Kingdom, The Netherlands, Spain, and the Czech Republic [7]. However, despite increasing interest and need driving native plant restoration, little information is available on the microbial communities involved in essential soil processes such as the nitrogen cycle in these abandoned cropland ecosystems.

The nitrogen cycle is an important process in natural ecosystems and traditional agriculture; it supplies nitrogen—essential for plant growth—to arable soil through nitrogen fixation. Moreover, the nitrogen cycle plays a major role in climate change. Nitrification and denitrification mediated by this process might lead to nitrate leaching from the soil, because nitrogen-containing nutrients are converted to gaseous products such as nitrous oxide (N_2_O) or nitrogen (N_2_). N_2_O is a greenhouse gas that contributes more to atmospheric warming than carbon dioxide [8].

The nitrogen cycle has been investigated in diverse soil types, such as those present in agricultural fields [9], paddies [10], and forests [11]. However, these studies focused on either areas with stable vegetation populations or those with a single vegetation type. In abandoned cropland, vegetation gradually changes with time, thus imposing different demands on growth-limiting factors such as the availability of nitrogen. In turn, these fluctuating demands influence the microbial community that contributes to the nitrogen cycle. Therefore, this study aimed to (1) reconstruct functional microbial communities involved in key processes of the inorganic nitrogen cycle, and (2) link these results to the abiotic and biotic properties of cropland soils that were abandoned for different periods. For this purpose, we used real-time polymerase chain reaction (PCR) to determine the abundance of bacterial genes that encode nitrogenase (*nifH*), ammonia monooxygenase (*amoA* of bacteria and archaea), nitrite reductase (*nirK* and *nirS*), and nitrous oxide reductase (*nosZ*). We further determined whether the distribution of these genes correlated with soil and plant characteristics.

## Materials and Methods

### Ethics statement

No specific permissions were required for conducting field survey in this area. The study was conducted on public land and complied with all relevant regulations. Our field studies did not involve any endangered or protected plant species.

### Site description and soil sampling

**Fig 1:**
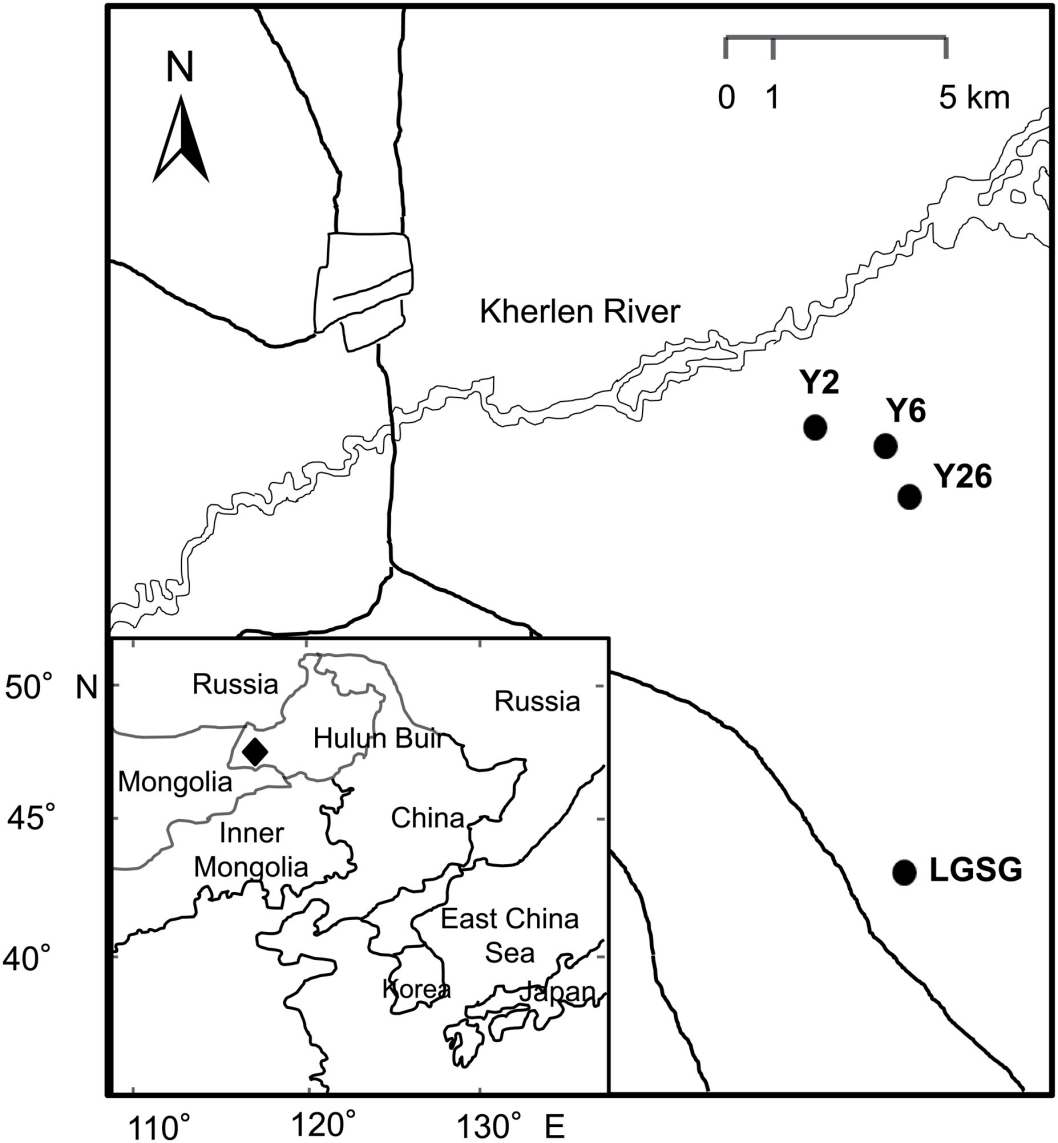
Study area (◆) and sediment sampling sites (●) in the Hulun Buir grassland. Note: Reprinted from [12] under a CC BY license, with permission from Huhe, original copyright 2014.

The study area is located in the semi-arid areas of the Hulun Buir grassland (115°31′–126°04′ E, 47°05′–53°20′ N) in northeastern Inner Mongolia, China (Fig 1). The sampling sites used in this study were documented previously [12], as three abandoned croplands and a light-grazing steppe grassland (LGSG) that had a grazing intensity of approximately 1.4 sheep·ha^−1^. The three croplands were abandoned for 2, 6, and 26 years (Y2, Y6, and Y26), respectively, and the LGSG served as the control (Fig 1). Study site characteristics are provided in Table 1.

**Table 1:** Characteristics of the study sites.

Site code	Abandonment time (y)	Coordinates		Elevation (m)	AAP^a^ (mm)	AAT^b^ (°C)	Soil texture
		Latitude	Longitude				
Y2	2	48° 38′ 43′′ N	116° 57′ 56′′ E	545	213	1.6	Sandy loam
Y6	6	48° 38′ 50′′ N	117° 00′ 48′′ E	550	213	1.6	Sandy loam
Y26	26	48° 38′ 45′′ N	117° 01′ 56′′ E	545	213	1.6	Sandy loam
LGSG	―	48° 33′ 06′′ N	117° 00′ 35′′ E	568	213	1.6	Sandy loam

^a^ Average annual precipitation.

^b^ Average annual temperature.

Plant surveying and soil sampling were conducted in August 2011. Sites were selected based on whether they possessed similar topography (flat) and vegetation. Each site contained 5 replicates in a randomized plot (1 × 1 m) design. In each plot, the soil samples were collected from 5 randomly selected points (0–10 cm deep) and mixed into one sample. Each soil sample was split into two equal parts: one for DNA isolation (stored at -20°C), and the other for chemical analyses (stored at -4°C). The mean temperature and precipitation (2000 to 2009) were obtained from online data reported by Matsuura and Willmott [13].

### DNA extraction

DNA from soil subsamples was extracted using the Powersoil DNA extraction kit (MoBio Laboratories, Carlsbad, CA), following manufacturer protocol. Three replicates of 0.5 g soil per plot (4 sites × 5 replicates; i.e., 20 plots) were extracted to reduce potential bias from soil microsite heterogeneity. The extracted DNA was examined on 1.0% agarose gels after electrophoresis. The quality and quantity of DNA extracts were checked using a SmartSpec Plus spectrophotometer (Bio-Rad Laboratories, US). Samples were pooled and stored at -20°C.

### Real-time quantitative PCR assay

Real-time quantitative PCR was used to quantify the population sizes of nitrogen fixing (*nifH*) and ammonia oxidizing bacteria (AOB), ammonia-oxidizing archaea (AOA), denitrifiers (*nirK* and *nirS*), and nitrous oxide reducers (*nosZ*) in the DNA of soil samples extracted from three different abandoned croplands and the control LGSG soil.

PCR was performed using primers listed in S1 Table by using the Power SYBR Green PCR master mix (Applied Biosystems), according to manufacturer protocol. The PCR was run using the Applied Biosystems StepOne Plus 96-well real-time PCR system. The composition of each reaction mixture is listed in S2 Table. All PCRs started with an enzyme activation step performed at 95°C for 10 min. The subsequent thermal profile was different for each gene (S1 Table).

The specificity of the amplified products was confirmed by observing a single melting peak and the presence of a unique band of the expected size on a 2% agarose gel stained with ethidium bromide. The possible inhibitory effects on PCRs caused by humic substances were investigated by first determining the optimal dilution for each DNA extract, and three subsamples were used. Serial dilutions of plasmids harboring the respective genes ranging from 10^1^–10^7^ copies·μl^–1^ served as controls (sources of standards are shown in S1 Table). A plasmid containing the archaeal *amoA* gene (clone LGSGa2) was used for quantification, following the methods described by Kelly et al. [14].

From a BLAST [15] analysis of the GenBank nucleotide sequence database, clone LGSGa2 was identified as archaeal *amoA*. The sequence of LGSGa2 was deposited in GenBank under accession number AB740225. The gene copy numbers were normalized to grams of dry soil. Amplification efficiencies were calculated using the formula Eff = [10(^-1/slope^) - 1] and accounted for 98.5% of *nifH* genes, 98.8% of archaeal *amoA* genes, 93.4% of bacterial *amoA* genes, 92.0% of *nirK* genes, 98.2% of *nirS* genes, and 93.7% of *nosZ* genes.

### Statistical analysis

Real-time PCR data were subjected to linear regression by using the statistical software program SPSS (version 19.0; SPSS, Inc., Chicago, IL, USA). The results of five replicates for each plot were averaged, and the variation with time was determined using regression analysis. Next, correlations between the abundance of each nitrogen-cycle gene and environmental factor were tested using Pearson’s product-moment correlations. Normality and homogeneity of variance were assessed using the Kolmogorov–Smirnov test and Levene’s test, respectively. Non-normal data were log-transformed before analysis. Pearson’s product-moment correlations with *P* values adjusted by the Benjamini–Hochberg method were used to control the false-discovery rate [16].

First, the distribution of microbial nitrogen-cycle gene abundance was determined using a principal component analysis (PCA). Next, redundancy analysis (RDA) was performed to determine the correlation between abundance distribution and environmental factors. RDA was used because a preliminary, detrended correspondence analysis indicated that the longest gradient was smaller than 3.0 [17]. PCA and RDA were performed using CANOCO software for Windows 4.5 (Biometris, Wageningen, The Netherlands).

## Results

### Quantification of nitrogen-cycle genes

**Fig 2:**
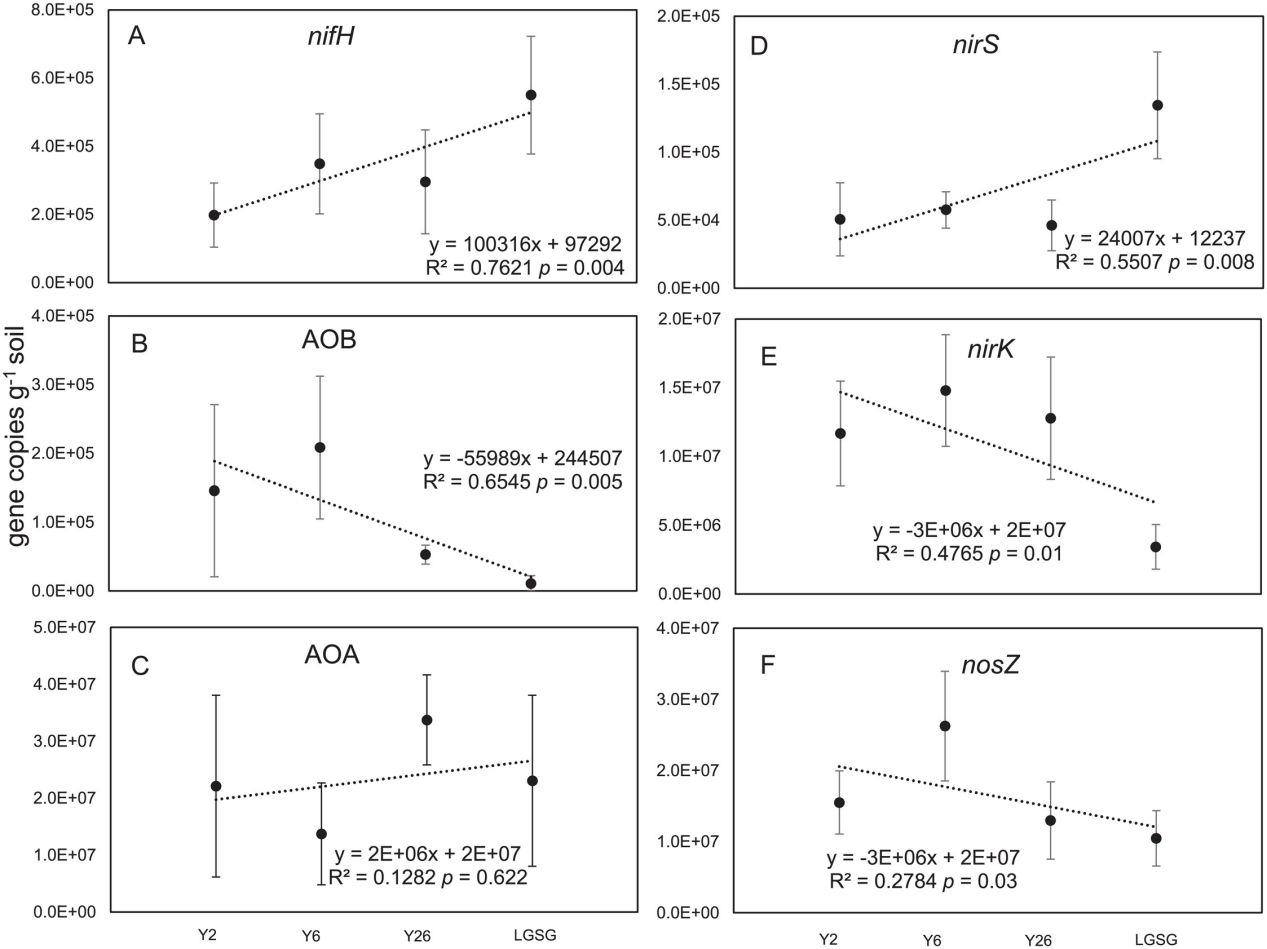
The results of linear regression between nitrogen-cycle gene copy numbers (log scale) and cropland abandonment period. (A), *nifH* gene; (B), AOB gene; (C), AOA gene; (D), *nirS* gene; (E), *nirK* gene; (F), *nosZ* gene. The regression lines are shown with R^2^.

Regression analysis revealed that cropland abandonment period significantly co-varied nitrogen-cycle gene abundance. With an increase in abandonment period, *NifH* and *nirS* copies also increased (Fig 2A and 2D respectively), whereas AOB, *nirK*, and *nosZ* copies decreased (Fig 2B, 2E and 2F, respectively). However, AOA gene copies did not change with increasing abandonment period (Fig 2C).

For *nifH*, the copy numbers at the LGSG site were approximately 1.5- to 2.7-fold higher than those of the abandoned croplands (Fig 2A and S3 Table).

AOB abundance and AOA abundance exhibited different patterns in abandoned croplands versus LGSG soil. The abundance of AOB genes in the abandoned cropland sites was approximately 5- to 10-fold higher than that in the LGSG (Fig 2B and S3 Table). The AOA gene numbers were approximately 10^3^-fold higher than AOB genes (Fig 2B and 2C and S3 Table).

The denitrification potential of the soil was investigated by quantifying two complementary genes encoding nitrite reductase (*nirS* and *nirK*) (Fig 2D and 2E and S3 Table). *NirS* copy numbers were the highest in LGSG soil than in the abandoned cropland soils, whereas *nirK* copy numbers were actually the lowest in LGSG soil. The abundance of *nirK* was approximately 1000-fold and 10-fold higher than that of *nirS* in the abandoned cropland soils and LGSG soil, respectively.

Finally, *nosZ* abundance was lower in the soil at the LGSG site than that at the abandoned cropland sites (Fig 2F and S3 Table).

### Effects of environmental variables on the abundance of nitrogen-cycle genes

**Fig 3:**
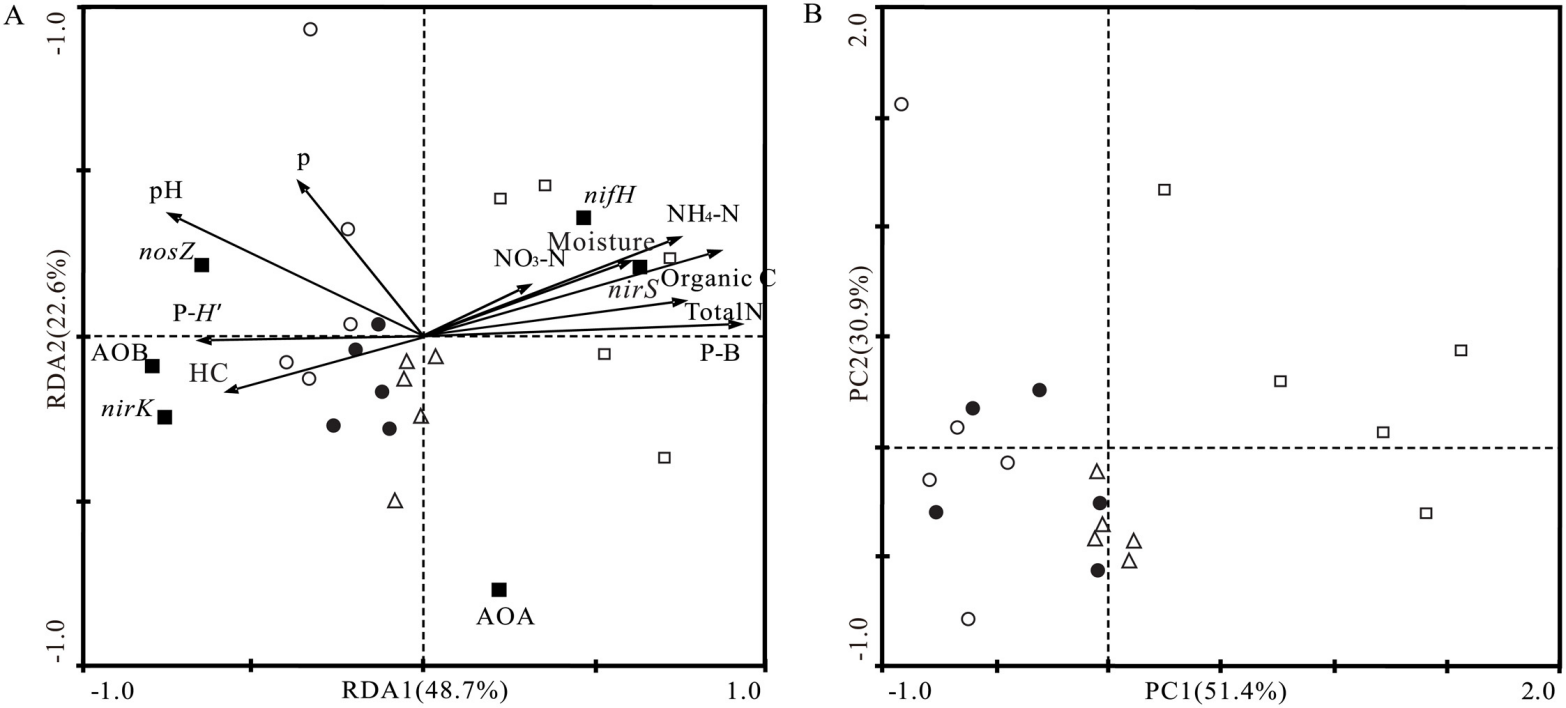
Relationships between nitrogen-cycle gene abundance and environmental variables based on redundancy analysis (A) and distribution of nitrogen-cycle gene abundance based on principle component analysis (B). Each site contained five replicates of a randomized plot (1 × 1 m) design as follows: cropland abandoned for 2 years, or Y2 (site 1, ●); cropland abandoned for 6 years, or Y6 (site 2, ○); cropland abandoned for 26 years, or Y26 (site 3, △); and the control light-grazing steppe grassland, or LGSG (site 4, □). Nitrogen-cycle genes are indicated by closed squares (■) and environmental variables are indicated by arrows.

The distribution analysis of nitrogen-cycle genes based on the RDA was consistent with the PCA results (Fig 3A and 3B). The first two canonical axes explained 71.3% and 82.3% of gene variance in the RDA and PCA, respectively. For the RDA, the two axes also explained 63.4% of variance in the species-environment relationship. The Pearson’s product-moment correlation and RDA results identifying a relationship between nitrogen-cycle gene abundance and environmental variables are shown in Table 2 and Fig 3A. The *nifH* and *nirS* copy numbers were positively correlated with NH_4_-N, organic C, total N, and plant biomass (P-B; *q* < 0.05), but negatively correlated with plant diversity (P-*H′*; *q* < 0.05). AOB, *nirK*, and *nosZ* showed significant positive relationships with pH and negative relationships with P-B (*q* < 0.05). AOB and *nirK* were also positively correlated with P-*H′* and hydraulic conductivity (HC; *q* < 0.05), and *nirK* was negatively correlated with NH_4_-N, organic C, and total N (*q* < 0.05). AOA copy numbers were not significantly correlated with any of the investigated environmental factors (*q* > 0.05).

**Table 2:** Changes in environmental variables compared to the abundance of microbial nitrogen cycle-related genes.

Abandoned cropland or significance parameter ^a^	pH	NO_3_-N (mg·kg^-1^)	NH_4_-N (mg·kg^-1^)	P (mg·kg^-1^)	Organic C (g·kg ^-1^)	Total N (g·kg^-1^)	Moisture (%)	P-*H′*	P-B	HC (×10^−3^ cm·s^-1^)
Abandoned cropland ^b^										
Y2	7.86 ± 0.17	3.46 ± 0.24	1.63 ± 0.43	11.98 ± 3.91	5.89 ± 0.65	0.65 ± 0.05	8.41 ± 0.17	1.59 ± 0.41	50.1 ± 8.56	2.94 ± 0.06
Y6	8.42 ± 0.41	3.82 ± 0.47	0.72 ± 0.15	12.65 ± 1.98	9.25 ± 0.41	0.98 ± 0.21	9.26 ± 0.15	1.67 ± 0.23	20.61 ± 4.52	2.93 ± 0.04
Y26	7.32 ± 0.12	3.52 ± 0.12	1.10 ± 0.37	12.25 ± 1.52	7.58 ± 0.44	0.77 ± 0.08	10.58 ± 1.11	1.85 ± 0.19	72.21 ± 11.5	2.75 ± 0.05
LGSG	6.18 ± 0.15	4.25 ± 0.12	4.64 ± 0.45	23.96 ± 6.12	24.56 ± 3.91	2.8 ± 1.82	12.25 ± 1.51	0.94 ± 0.25	142.41 ± 5.72	2.73 ± 0.02
Correlation (*q*^c^)										
with *nifH* copy number	NS	NS	++	++	++	++	++	--	++	NS
with AOB copy number	++	NS	NS	NS	NS	NS	NS	++	—	++
with AOA copy number	NS	NS	NS	NS	NS	NS	NS	NS	NS	NS
with *nirK* copy number	++	NS	--	NS	--	--	NS	++	--	++
with *nirS* copy number	--	NS	++	NS	++	++	NS	--	++	NS
with *nosZ* copy number	++	NS	NS	++	NS	NS	NS	NS	--	NS

^a^ All environmental variables are shown and include pH, NO_3_-N, NH_4_-N, available phosphorus (P), organic carbon (organic C), total nitrogen (total N), soil moisture, plant diversity (P-*H*′), plant biomass (P-B), and hydraulic conductivity (HC).

^b^ Y2, Y6, and Y26 indicate the number of years that the fields were abandoned (2, 6, and 26 years, respectively). LGSG, light-grazing steppe grassland. The values shown for management factors represent the mean ± standard error.

^c^ *q*, Pearson’s product-moment correlation coefficient; NS, not significant; ++/--, significant positive or negative correlation, *q* < 0.05.

## Discussion

In this study, we analyzed the relationship between abandoned cropland soils and the abundance of microbial genes that mediate the nitrogen cycle.

We found significant changes in nitrogen-cycle gene abundance with increasing abandonment period (Fig 2A, *p* = 0.004; Fig 2B, *p* = 0.005; Fig 2D, *p* = 0.008; Fig 2E, *p* = 0.01; Fig 2F, *p* = 0.03). These patterns were likely because of the changes in soil properties and vegetation characteristics that occurred with increasing abandonment period.

With an increase in the abandonment period, *nifH* abundance also increased (Fig 2A) and was positively correlated with organic C (Table 2 and Fig 3A), which indicates the importance of carbohydrate sources for N_2_ fixation activity, an energetically expensive process that requires large amounts of reducing equivalents [18].

Organic matter serves as a long-term, slow-release storehouse of nitrogen, phosphorus, and sulfur [19]. Therefore, we found that *nifH* abundance was positively correlated with both P and total N. The large amounts of ATP required for nitrogen fixation suggests that increased phosphorus can also increase nitrogen-fixing activity [20]. However, interestingly, in contrast to our results (Fig 2A and Table 2), Coelho et al. [21, 22] found that 30% more free-living diazotrophs were extracted from soils containing low levels (12 kg N·hectare^-1^), rather than high levels (120 kg N·hectare^-1^), of nitrogen-treated fertilizer. This difference in these findings indicates that the free-living diazotrophs in nitrogen fixation might vary among different regions, because the soil microbial community is not affected by a single environmental factor, but co-regulated by various environmental factors.

As expected, *nifH* abundance was positively correlated with NH_4_-N; however, AOB abundance was negatively correlated with NH_4_-N (Table 2). This might be because P-*H′* inhibited the nitrification process. Because Pearson’s product-moment correlations and RDA results suggest that ammonia-oxidizing bacteria were strongly influenced by P-*H′*, such a pattern indicates that these bacteria might be more susceptible to the different root exudates from microbe-specific plant defense and growth responses [23]. In the LGSG control soil, AOB abundance was lower than that in abandoned croplands. This could be caused by the high competition for free ammonia between plants and microbes in the LGSG, a process also documented in grasslands [24] and other terrestrial ecosystems [25].

Nicol et al. [26] found that, under low pH conditions, AOB abundance was about 10^3^-fold lower than the AOA abundance, but increased with increasing pH. In the present study, AOB abundance was also correlated positively with pH. In contrast, gene copy numbers of archaeal ammonia oxidizers were constant at all sites, and no environmental factors significantly influenced them (Table 2 and Fig 3A), indicating that the archaea adapt to changing environmental conditions. Thus, our findings corroborate those of previous studies [27], indicating the dominance of archaeal ammonia oxidizers over their bacterial counterparts in agricultural and grassland soils.

The *nirS* and *nirK* denitrifiers increased and decreased with increasing abandonment period, respectively. The *nirK*-type denitrifiers were affected by P-*H′*. Similar to our AOB results, this indicates that *nirK*-type denitrifiers might be more readily affected by root exudate amount and composition, consistent with the findings of Bremer et al. [28]. In addition, the abundance of *nirS*-type denitrifiers correlated positively with organic C and P-B, whereas correlated negatively with pH, suggesting that these denitrifiers are adapted to conditions with low pH [29–32]. Additionally, AOB and *nirK* were positively correlated with HC, suggesting that the abundance of AOB and *nirK* might be affected by the balance between macropores and micropores. This could be because the soil texture and hydraulic conductivity were closely correlated, and soil texture is known to influence the balance between macropores and micropores [33].

Unlike *nirK*, *nirS* gene copies were significantly fewer in our study area. Specifically, *nirK* abundance was approximately 1 to 3 orders of magnitude higher than *nirS* abundance both at the control LGSG site and the abandoned cropland sites, suggesting that *nirK-*type denitrifiers are important in this region. A dominance of *nirK* over *nirS* in different soils has also been reported in previous studies [10, 34].

In this study, *nosZ* abundance showed a decreasing trend with increasing abandonment periods (Fig 2F). Of all factors that might affect *nosZ* denitrifier abundance, pH was thought to be the most important (Table 2 and Fig 3A). Similarly, other studies have reported that pH strongly affects the *nosZ* denitrifier community in agricultural [35, 36], forested upland, and wetland soils [37].

With the exception of nitrifying archaea and nitrous oxide-reducing bacteria, the genotypic abundance of microbial communities involved in the nitrogen cycle clearly differed (about 2- to 10-fold) between abandoned cropland and LGSG soils. This outcome might likely be attributed to the nutrient deficiency caused by continuous cultivation. Consistent with this idea, plant biomass was found to be the most abundant at the LGSG site, where elevated contents of organic C, P, and total N were observed (Table 2). In contrast, the contents of these compounds were not remarkably different between the three abandoned cropland sites, which could explain the very little differences in the effect of these soils (Table 2), despite clear differences in plant biomass. Therefore, while plant biomass might shift more rapidly in response to environmental change, soil nutrient pools, which reflect a history of agricultural land use, can persist for decades [38].

Although regression analysis revealed that nitrogen-cycle gene abundances differed with abandonment time, a clear difference could be noted between the abundance patterns in abandoned cropland versus LGSG (Fig 3A and 3B). This outcome suggests that, despite the lack of cultivation for over a quarter-century, nitrogen-cycle genes were still not completely recovered. The distribution of nitrogen-cycle genes was also strongly affected by plant biomass and plant diversity in our study area, reflecting the ability of plants to regulate nitrogen cycle microbial communities. The interpretation of our data is limited by the lack of replication in other abandoned fields. While logistical constraints (lack of suitable abandoned cropland) prevented us from expanding this study, four experimental sites were very similar in environmental parameters (soil texture, temperature, precipitation and elevation; Table 1), but differed considerably in vegetation characteristics, and five plots in each experimental site were very similar in vegetation characteristics [12]. Thus, mitigating the problem of pseudoreplication is possible since the effect of vegetation on nitrogen-cycle gene abundance is more likely to be similar among the five plots at each experimental site. However, this still does not address the pseudo-replication phenomenon, therefore, the results could be affected by one or more unmeasured environmental parameters that might vary between the region containing the steppe grassland plot and the region containing the abandoned croplands.

To better understand the complex interrelationship between nonsymbiotic plants and microbes, future studies need to focus on the effect of different plant species on the soil microbial community (for instance, by determining the effect of interspecific variation in root exudates on microbial gene abundance). Furthermore, replicating our study in successive seasons and on additional sites might lead to the elucidation of whether our current results can be generalized to all abandoned cropland ecosystems in semi-arid grasslands.

## Supporting Information

S1 File Permission from the original copyright holder. (DOCX)

S2 File References. (DOCX)

S1 Table Primer sets and thermal profiles used for the quantitative polymerase chain reaction analyses. (DOCX)

S2 Table Reaction components of Mastermix assay (25 μL used for quantitative polymerase chain reaction analyses. (DOCX)

S3 Table Copy numbers of the nitrogen-cycle genes in steppe grassland and abandoned cropland soils. (DOCX)
