# Supplementary material for: Microbial Nitrogen-Cycle Gene Abundance in Soil of Cropland Abandoned for Different Periods
Source: PLoS One. 2016 May 3;11(5):e0154697. doi: 10.1371/journal.pone.0154697 (PMC4854452; doi:10.1371/journal.pone.0154697)
Supplement: S1 File — (DOCX) [file pone.0154697.s001.docx]

**S1 File. Permission from the original copyright holder**

Huhe and colleagues permit for the open-access journal PLOS ONE to publish Fig. 1 under the Creative Commons Attribution License (CCAL) CC BY 4.0, and we are aware that this license allows unrestricted use and distribution, even commercially, by third parties.
