# Supplementary material for: Microbial Nitrogen-Cycle Gene Abundance in Soil of Cropland Abandoned for Different Periods
Source: PLoS One. 2016 May 3;11(5):e0154697. doi: 10.1371/journal.pone.0154697 (PMC4854452; doi:10.1371/journal.pone.0154697)
Supplement: S2 File — (DOCX) [file pone.0154697.s002.docx]

**S2 File. References**

1. Henry S, Baudoin E, Lopez-Gutierrez JC, Martin-Laurent F, Baumann A, Philippot L (2004) Quantification of denitrifying bacteria in soils by *nirK* gene targeted real-time PCR. J Microbiol Meth 59: 327-335.
2. Henry S, Bru D, Stres B, Hallet S, Philippot L (2006) Quantitative detection of the *nosZ* gene, encoding nitrous oxide reductase, and comparison of the abundances of 16S rRNA, *narG*, *nirK*, and *nosZ* genes in soils. Appl Environ Microbiol72: 5181-5189.
3. Leininger S, Urich T, Schloter M, Schwark L, Qi J, Nicol GW, et al (2006) Archaea predominate among ammonia-oxidizing prokaryotes in soils. Nature442: 806-809.
4. Michotey V, Mejean V, Bonin P (2000) Comparison of methods for quantification of cytochrome cd(1)-denitrifying bacteria in environmental marine samples.Appl Environ Microbiol66: 1564-1571.
5. Rosch C, Mergel A, Bothe H (2002) Biodiversity of denitrifying and dinitrogen-fixing bacteria in an acid forest soil. Appl Environ Microbiol 68: 3818-3829.
6. Rotthauwe JH, Witzel KP, Liesack W (1997) The ammonia monooxygenase structural gene *amoA* as a functional marker: molecular fine-scale analysis of natural ammonia-oxidizing populations. Appl Environ Microbiol63: 4704-4712.
7. Schauss K, Focks A, Leininger S, Kotzerke A, Heuer H, Thiele-Bruhn S et al (2009) Dynamics and functional relevance of ammonia-oxidizing archaea in two agricultural soils. Environ Microbiol11: 446-456.
8. Throback IN, Enwall K, Jarvis A, Hallin S (2004) Reassessing PCR primers targeting nirS, *nirK* and *nosZ* genes for community surveys of denitrifying bacteria with DGGE. FEMS MicrobiolEcol 49: 401-417.
