# Supplementary material for: Microbial Nitrogen-Cycle Gene Abundance in Soil of Cropland Abandoned for Different Periods
Source: PLoS One. 2016 May 3;11(5):e0154697. doi: 10.1371/journal.pone.0154697 (PMC4854452; doi:10.1371/journal.pone.0154697)
Supplement: S1 Table — (DOCX) [file pone.0154697.s003.docx]

**S1 Table. Primer sets and thermal profiles used for the qPCR analyses.**

| Gene | Primer | Reference | Amplicon size (bp) | Source of standard | Thermal cycling profile | Nomber of cycles |
| --- | --- | --- | --- | --- | --- | --- |
| *nifH* | nifHF  nifHR | [5] | 458 | *Azospirillumbrasilense* ATCC 29729 | 95°C/45 s, 55°C/45 s, 72°C/45 s | 40 |
| Archaeal *amoA* | amo19F  CrenamoA616r48x | [3]  [7] | 624 | Plasmid clone LGSGa2 | 94°C/45 s, 55°C/60 s, 72°C/60 s | 40 |
| Bacterial *amoA* | amoA1F  amoA2R | [6] | 500 | *Nitrosomonaseuropaca* ATCC 19718 | 94°C/60 s, 60°C/60 s, 72°C/60 s | 40 |
| *nirK* | nirK876C  nirK1040 | [1] | 164 | *Sinorhizobiummeliloti*1021 | 95°C/30 s, 63°C-58°C/30 s, 72°C/30 s  95°C/15 s, 58°C/30 s, 72°C/30 s | 6*  40 |
| *nirS* | cd3af  R3cd | [4]  [8] | 413 | *Pseudomonas aeruginosa* PAO1 | 94°C/60 s, 57°C/60 s, 72°C/60 s | 40 |
| *nosZ* | nosZ2F  nosZ2R | [2] | 267 | *Pseudomonas aeruginosa* PAO1 | 95 °C/30 s, 65-60 °C/30 s, 72 °C/30 s  95 °C/15 s, 60 °C/15 s, 72 °C/30 s | 6*  40 |

*** Touch down**
