# Supplementary material for: Microbial Nitrogen-Cycle Gene Abundance in Soil of Cropland Abandoned for Different Periods
Source: PLoS One. 2016 May 3;11(5):e0154697. doi: 10.1371/journal.pone.0154697 (PMC4854452; doi:10.1371/journal.pone.0154697)
Supplement: S2 Table — (DOCX) [file pone.0154697.s004.docx]

**S2 Table. Reaction components of Mastermix assay (25 µl) used for qPCR analyses.**

| Target gene | Amt (μl) of assay component | | | | | | |
| --- | --- | --- | --- | --- | --- | --- | --- |
|  | 2× Power SYBR green | 3% BSA | Primer (10 pmol μl^−1^) | | Dimethyl sulfoxide | DNA (1-5 ng µL^−1^) | PCR water |
|  |  |  | Forward | Reverse |  |  |  |
| *nifH* | 12.5 | 0.5 | 0.30 | 0.30 |  | 2 | 9.4 |
| Archaeal*amoA* | 12.5 | 0.5 | 0.75 | 0.75 |  | 2 | 8.5 |
| Bacterial*amoA* | 12.5 | 0.5 | 0.50 | 0.50 |  | 2 | 9.0 |
| *nirK* | 12.5 | 0.5 | 0.50 | 0.50 | 0.625 | 2 | 8.375 |
| *nirS* | 12.5 | 0.5 | 0.50 | 0.50 | 0.625 | 2 | 8.375 |
| *nosZ* | 12.5 | 0.5 | 0.50 | 0.50 | 0.625 | 2 | 8.375 |
