# Supplementary material for: Microbial Nitrogen-Cycle Gene Abundance in Soil of Cropland Abandoned for Different Periods
Source: PLoS One. 2016 May 3;11(5):e0154697. doi: 10.1371/journal.pone.0154697 (PMC4854452; doi:10.1371/journal.pone.0154697)
Supplement: S3 Table — (DOCX) [file pone.0154697.s005.docx]

**S3 Table.** **Copy numbers of the nitrogen-cycle genes in steppe grassland and abandoned cropland soils.**

| Abandoned Cropland or gene copy numbers | Avg. no of copies of nitrogen cycle genes/g of soil^a^ | | | | | |
| --- | --- | --- | --- | --- | --- | --- |
|  | *nifH* | AOB | AOA | *nirS* | *nirK* | *nosZ* |
| Y2 | 9.85E+06±3.89E+06 | 1.46E+05 ± 1.25E+05 | 2.21E+07 ± 1.02E+07 | 5.07E+04 ± 2.68E+04 | 1.17E+07 ± 3.81E+06 | 1.55E+07 ± 4.45E+06 |
| Y6 | 1.81E+07±6.52E+06 | 2.09E+05 ± 1.04E+05 | 2.57E+07 ± 1.56E+07 | 5.76E+04 ± 1.34E+04 | 1.48E+07 ± 4.07E+06 | 2.30E+07 ± 7.71E+06 |
| Y26 | 1.35E+07±7.98E+06 | 5.29E+04 ± 1.40E+04 | 3.37E+07 ± 7.91E+06 | 4.62E+04 ± 1.86E+04 | 1.28E+07 ± 4.46E+06 | 1.30E+07 ± 5.42E+06 |
| LGSG | 3.12E+07±7.66E+06 | 1.09E+04 ± 1.13E+04 | 3.50E+07 ± 1.60E+07 | 1.35E+05 ± 3.93E+04 | 3.42E+06 ± 1.63E+06 | 1.05E+07 ± 3.90E+06 |

^a^ The values shown represent the mean ± standard error.
